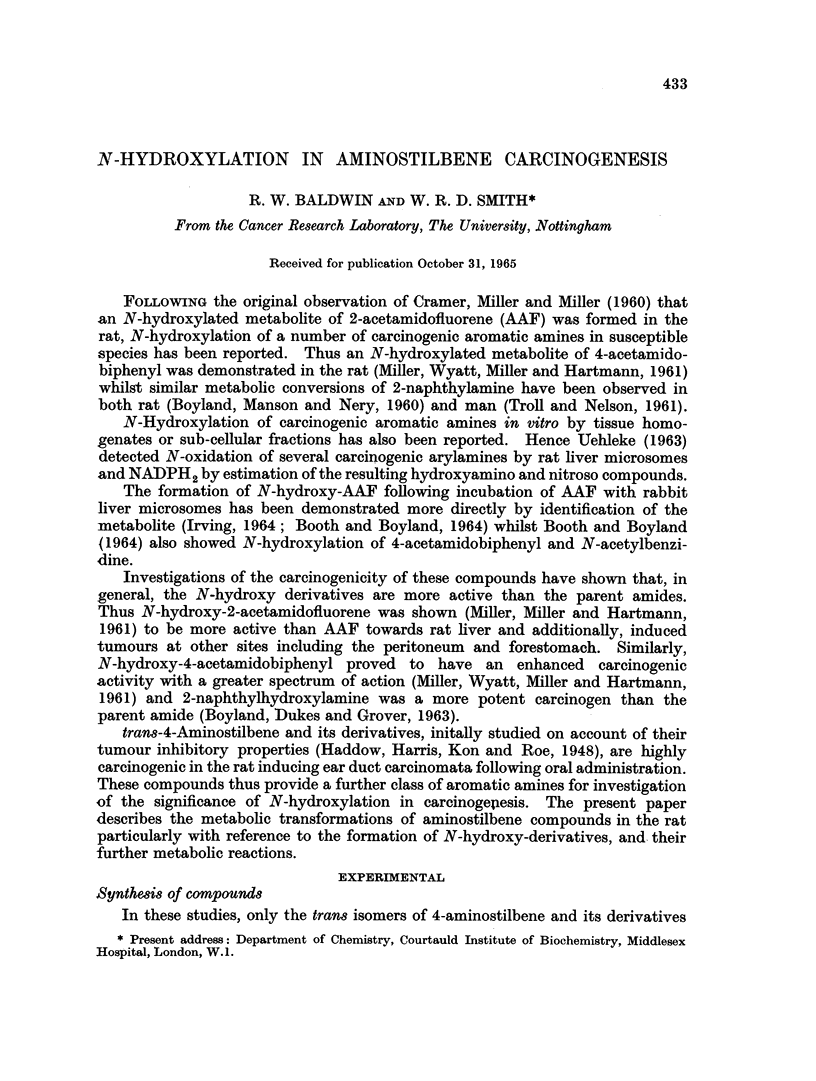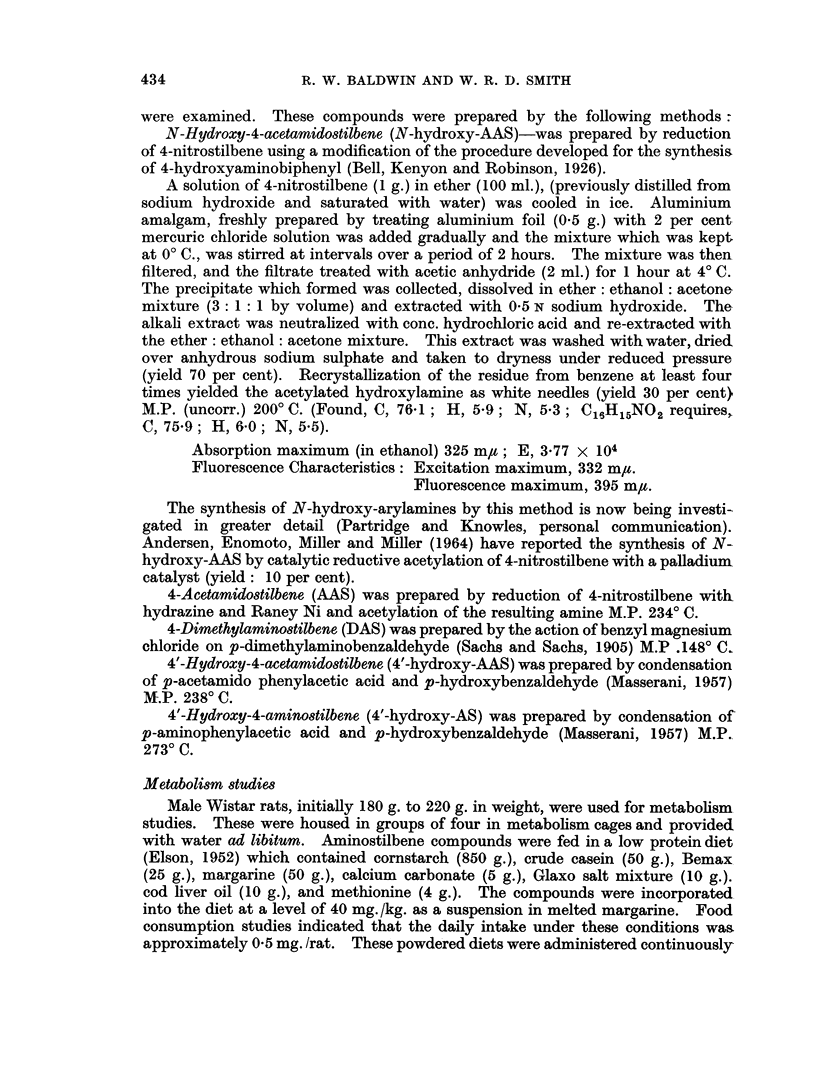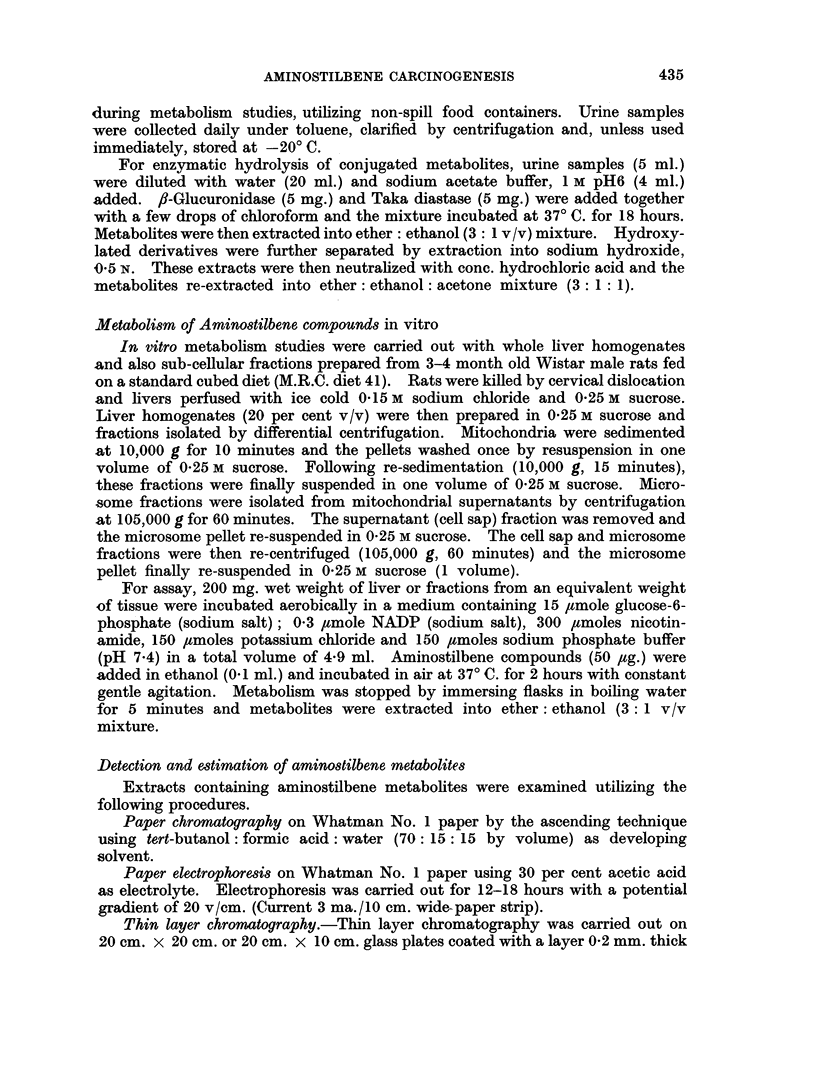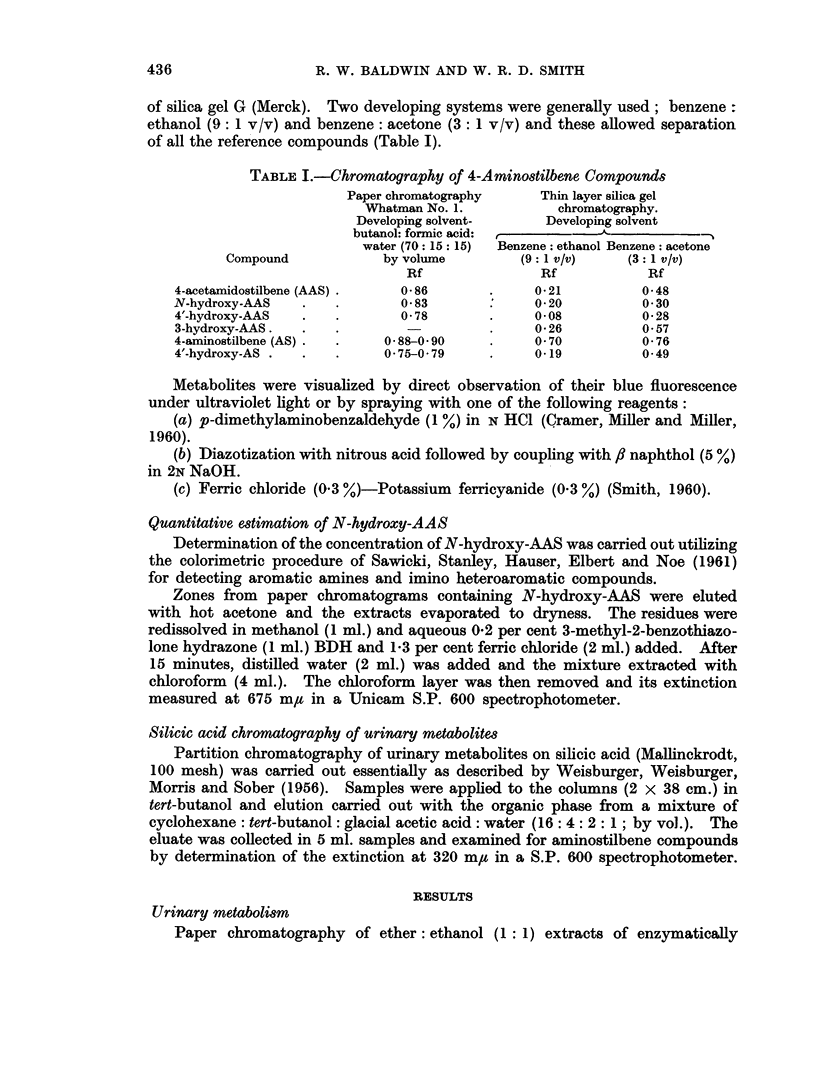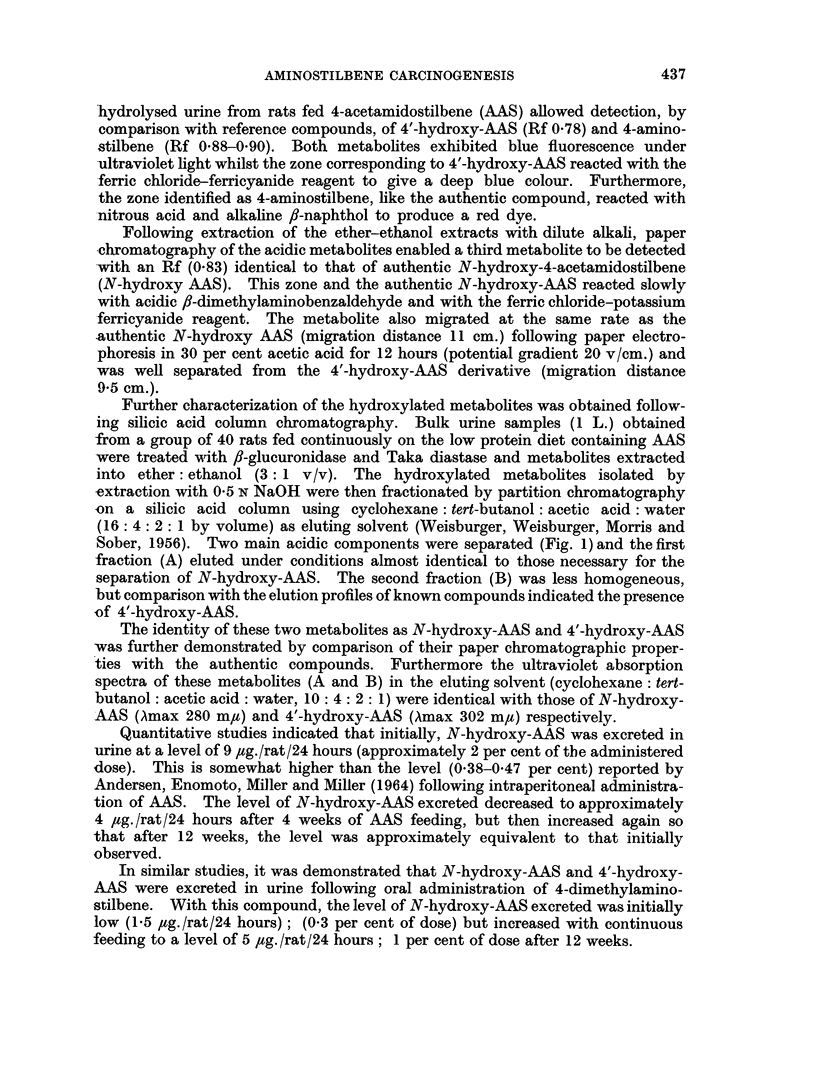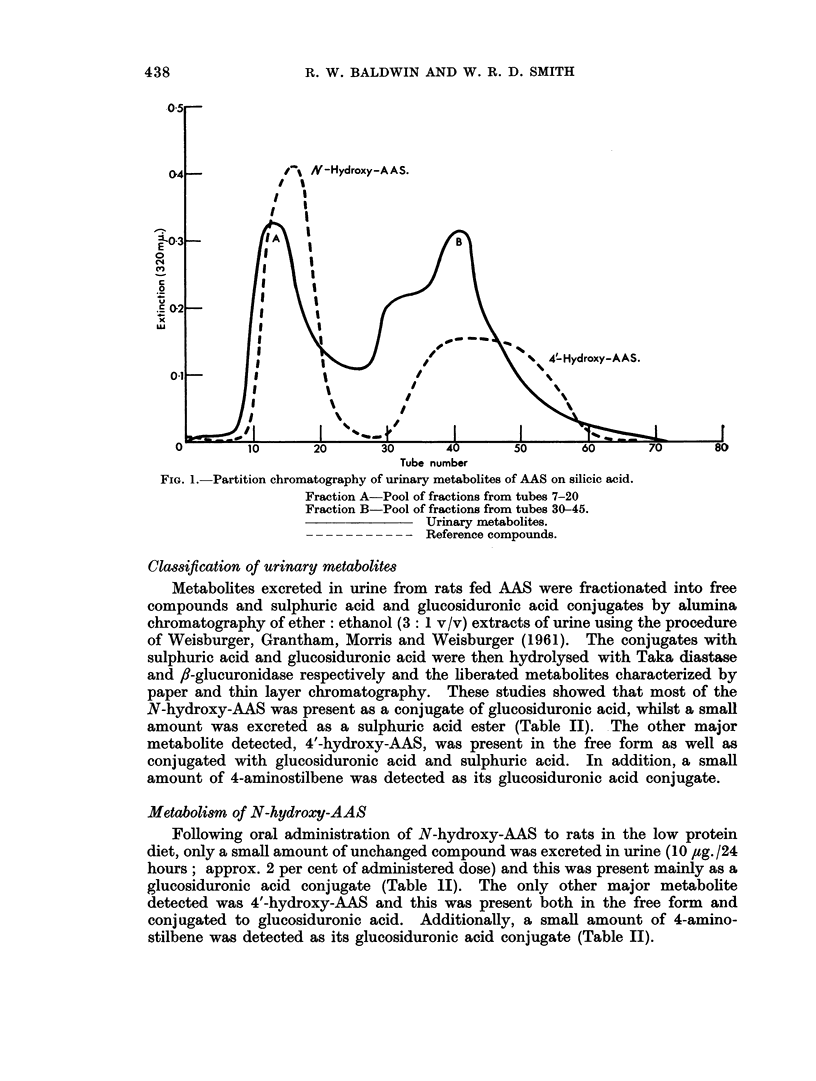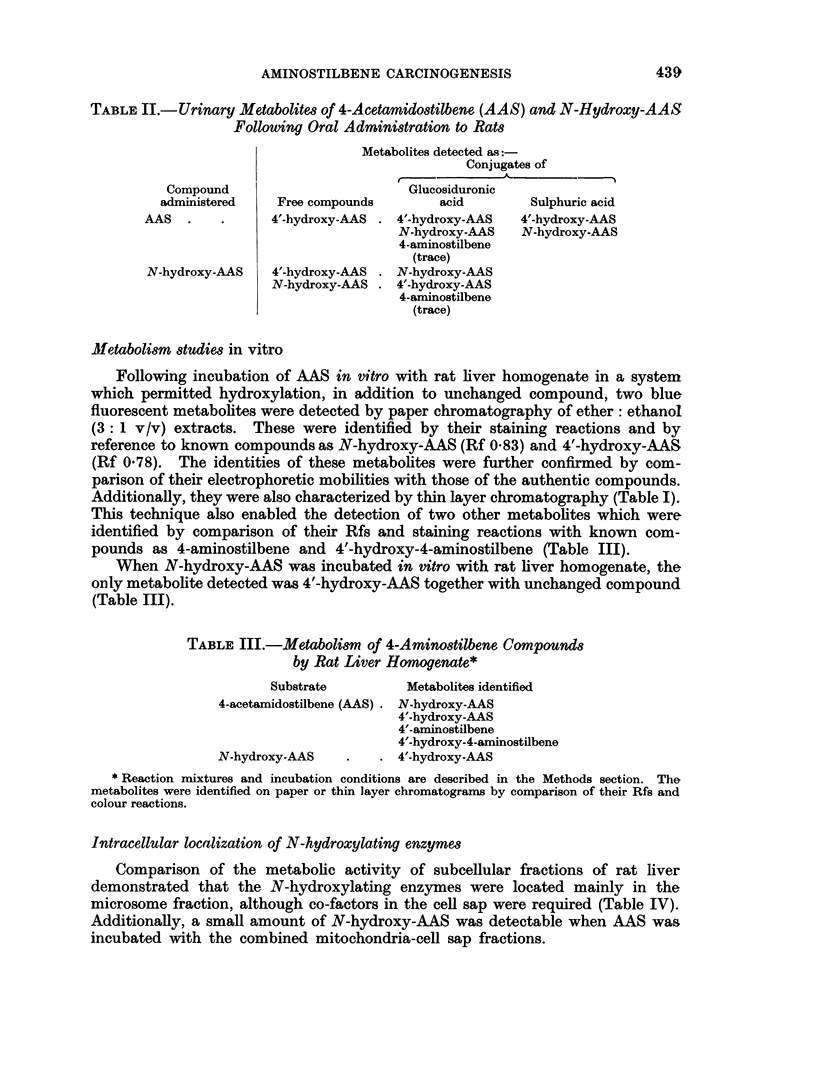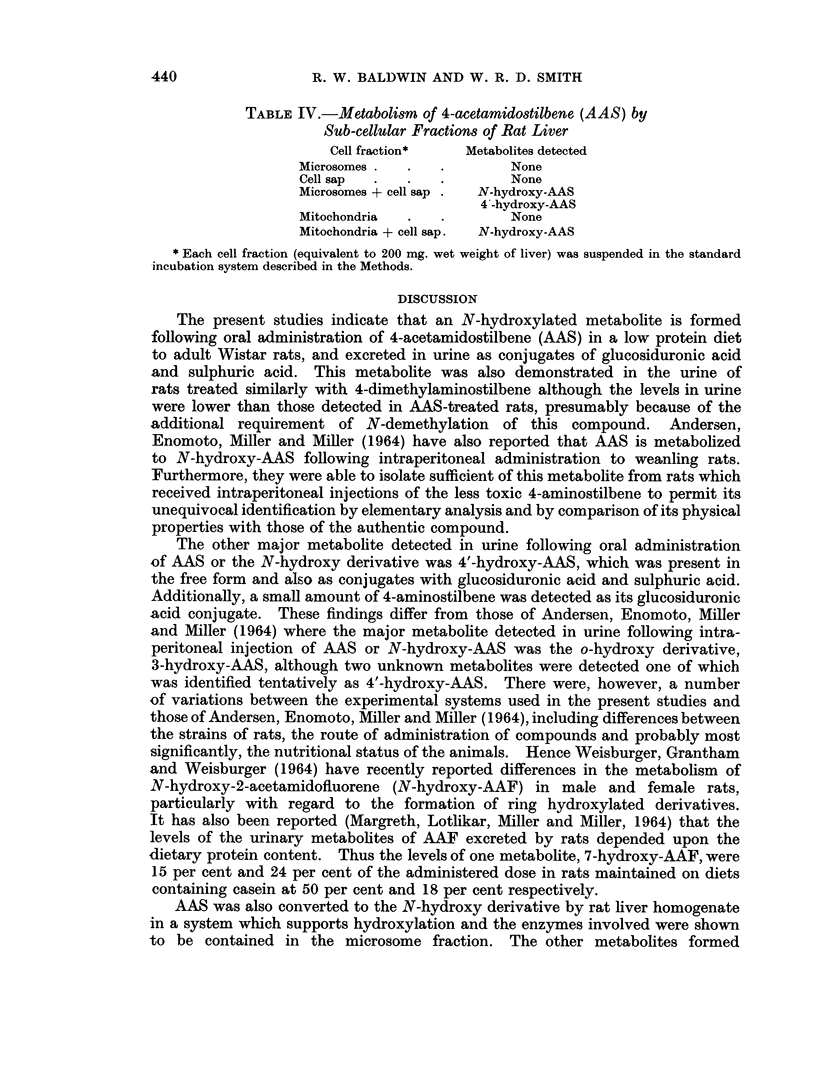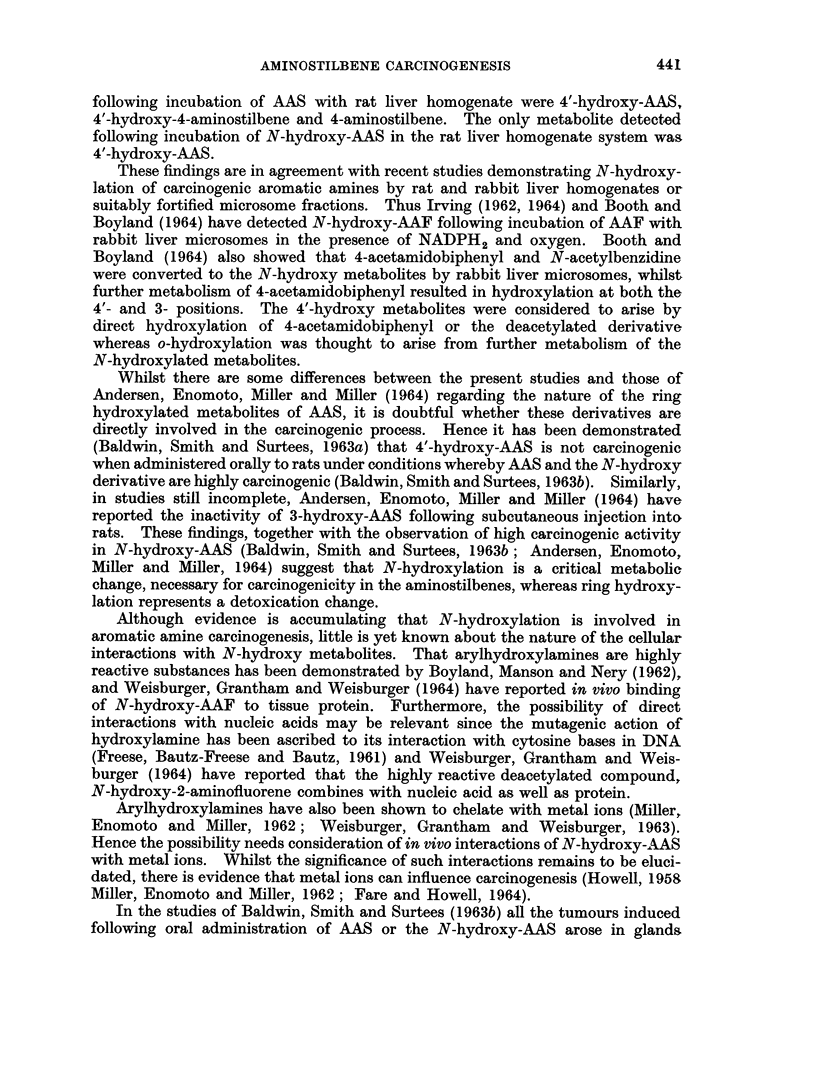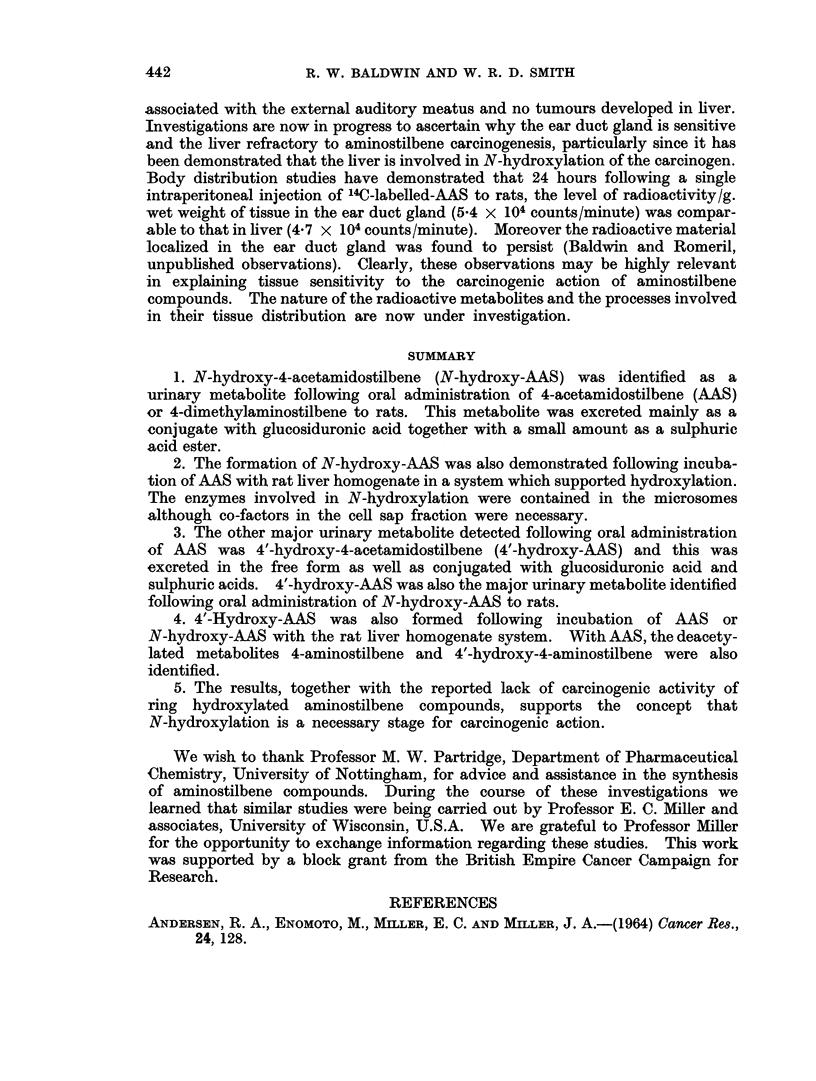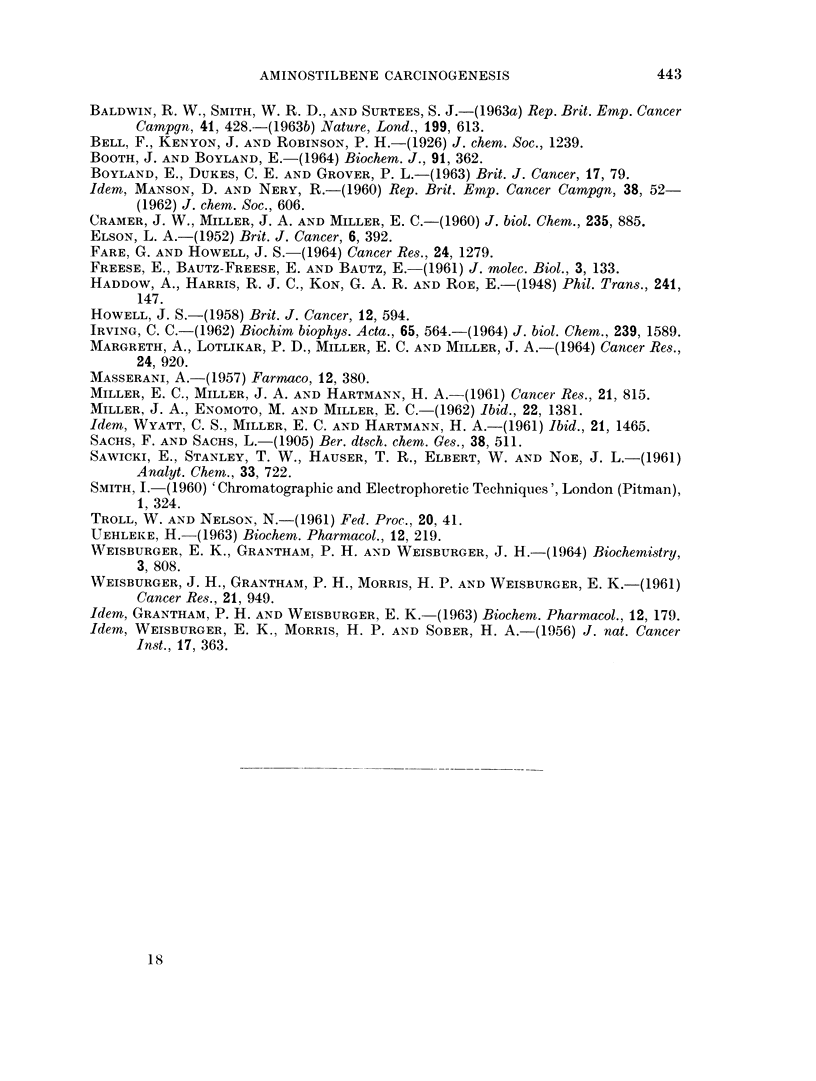# N-Hydroxylation in Aminostilbene Carcinogenesis

**DOI:** 10.1038/bjc.1965.52

**Published:** 1965-06

**Authors:** R. W. Baldwin, W. R. D. Smith


### $N$-HYDROXYLATION IN AMINOSTILBENE CARCINOGENESIS

R. W. BALDWIN AND W. R. D. SMITH*

*From the Cancer Research Laboratory, The University, Nottingham*

Received for publication October 31, 1965

FOLLOWING the original observation of Cramer, Miller and Miller (1960) that an $N$-hydroxylated metabolite of 2-acetamidofluorene (AAF) was formed in the rat, $N$-hydroxylation of a number of carcinogenic aromatic amines in susceptible species has been reported. Thus an $N$-hydroxylated metabolite of 4-acetamidobiphenyl was demonstrated in the rat (Miller, Wyatt, Miller and Hartmann, 1961) whilst similar metabolic conversions of 2-naphthylamine have been observed in both rat (Boyland, Manson and Nery, 1960) and man (Troll and Nelson, 1961).

$N$-Hydroxylation of carcinogenic aromatic amines *in vitro* by tissue homogenates or sub-cellular fractions has also been reported. Hence Uehleke (1963) detected $N$-oxidation of several carcinogenic arylamines by rat liver microsomes and $NADPH_2$ by estimation of the resulting hydroxyamino and nitroso compounds.

The formation of $N$-hydroxy-AAF following incubation of AAF with rabbit liver microsomes has been demonstrated more directly by identification of the metabolite (Irving, 1964; Booth and Boyland, 1964) whilst Booth and Boyland (1964) also showed $N$-hydroxylation of 4-acetamidobiphenyl and $N$-acetylbenzidine.

Investigations of the carcinogenicity of these compounds have shown that, in general, the $N$-hydroxy derivatives are more active than the parent amides. Thus $N$-hydroxy-2-acetamidofluorene was shown (Miller, Miller and Hartmann, 1961) to be more active than AAF towards rat liver and additionally, induced tumours at other sites including the peritoneum and forestomach. Similarly, $N$-hydroxy-4-acetamidobiphenyl proved to have an enhanced carcinogenic activity with a greater spectrum of action (Miller, Wyatt, Miller and Hartmann, 1961) and 2-naphthylhydroxylamine was a more potent carcinogen than the parent amide (Boyland, Dukes and Grover, 1963).

*trans*-4-Aminostilbene and its derivatives, initally studied on account of their tumour inhibitory properties (Haddow, Harris, Kon and Roe, 1948), are highly carcinogenic in the rat inducing ear duct carcinomata following oral administration. These compounds thus provide a further class of aromatic amines for investigation of the significance of $N$-hydroxylation in carcinogenesis. The present paper describes the metabolic transformations of aminostilbene compounds in the rat particularly with reference to the formation of $N$-hydroxy-derivatives, and their further metabolic reactions.

### EXPERIMENTAL

### *Synthesis of compounds*

In these studies, only the *trans* isomers of 4-aminostilbene and its derivatives

* Present address: Department of Chemistry, Courtauld Institute of Biochemistry, Middlesex Hospital, London, W.1.

were examined. These compounds were prepared by the following methods :

*N-Hydroxy-4-acetamidostilbene* (*N*-hydroxy-AAS)—was prepared by reduction of 4-nitrostilbene using a modification of the procedure developed for the synthesis of 4-hydroxyaminobiphenyl (Bell, Kenyon and Robinson, 1926).

A solution of 4-nitrostilbene (1 g.) in ether (100 ml.), (previously distilled from sodium hydroxide and saturated with water) was cooled in ice. Aluminium amalgam, freshly prepared by treating aluminium foil (0·5 g.) with 2 per cent mercuric chloride solution was added gradually and the mixture which was kept at 0° C., was stirred at intervals over a period of 2 hours. The mixture was then filtered, and the filtrate treated with acetic anhydride (2 ml.) for 1 hour at 4° C. The precipitate which formed was collected, dissolved in ether : ethanol : acetone mixture (3 : 1 : 1 by volume) and extracted with 0·5 N sodium hydroxide. The alkali extract was neutralized with conc. hydrochloric acid and re-extracted with the ether : ethanol : acetone mixture. This extract was washed with water, dried over anhydrous sodium sulphate and taken to dryness under reduced pressure (yield 70 per cent). Recrystallization of the residue from benzene at least four times yielded the acetylated hydroxylamine as white needles (yield 30 per cent) M.P. (uncorr.) 200° C. (Found, C, 76·1 ; H, 5·9 ; N, 5·3 ; $C_{16}H_{15}NO_2$ requires, C, 75·9 ; H, 6·0 ; N, 5·5).

Absorption maximum (in ethanol) 325 m$\mu$ ; E, 3·77 $\times$ $10^4$
Fluorescence Characteristics : Excitation maximum, 332 m$\mu$.
Fluorescence maximum, 395 m$\mu$.

The synthesis of *N*-hydroxy-arylamines by this method is now being investigated in greater detail (Partridge and Knowles, personal communication). Andersen, Enomoto, Miller and Miller (1964) have reported the synthesis of *N*-hydroxy-AAS by catalytic reductive acetylation of 4-nitrostilbene with a palladium catalyst (yield : 10 per cent).

*4-Acetamidostilbene* (AAS) was prepared by reduction of 4-nitrostilbene with hydrazine and Raney Ni and acetylation of the resulting amine M.P. 234° C.

*4-Dimethylaminostilbene* (DAS) was prepared by the action of benzyl magnesium chloride on *p*-dimethylaminobenzaldehyde (Sachs and Sachs, 1905) M.P .148° C.

*4′-Hydroxy-4-acetamidostilbene* (4′-hydroxy-AAS) was prepared by condensation of *p*-acetamido phenylacetic acid and *p*-hydroxybenzaldehyde (Masserani, 1957) M.P. 238° C.

*4′-Hydroxy-4-aminostilbene* (4′-hydroxy-AS) was prepared by condensation of *p*-aminophenylacetic acid and *p*-hydroxybenzaldehyde (Masserani, 1957) M.P. 273° C.

### *Metabolism studies*

Male Wistar rats, initially 180 g. to 220 g. in weight, were used for metabolism studies. These were housed in groups of four in metabolism cages and provided with water *ad libitum*. Aminostilbene compounds were fed in a low protein diet (Elson, 1952) which contained cornstarch (850 g.), crude casein (50 g.), Bemax (25 g.), margarine (50 g.), calcium carbonate (5 g.), Glaxo salt mixture (10 g.). cod liver oil (10 g.), and methionine (4 g.). The compounds were incorporated into the diet at a level of 40 mg./kg. as a suspension in melted margarine. Food consumption studies indicated that the daily intake under these conditions was approximately 0·5 mg./rat. These powdered diets were administered continuously during metabolism studies, utilizing non-spill food containers. Urine samples were collected daily under toluene, clarified by centrifugation and, unless used immediately, stored at −20° C.

For enzymatic hydrolysis of conjugated metabolites, urine samples (5 ml.) were diluted with water (20 ml.) and sodium acetate buffer, 1 M pH6 (4 ml.) added. β-Glucuronidase (5 mg.) and Taka diastase (5 mg.) were added together with a few drops of chloroform and the mixture incubated at 37° C. for 18 hours. Metabolites were then extracted into ether : ethanol (3 : 1 v/v) mixture. Hydroxylated derivatives were further separated by extraction into sodium hydroxide, 0·5 N. These extracts were then neutralized with conc. hydrochloric acid and the metabolites re-extracted into ether : ethanol : acetone mixture (3 : 1 : 1).

*Metabolism of Aminostilbene compounds* in vitro

*In vitro* metabolism studies were carried out with whole liver homogenates and also sub-cellular fractions prepared from 3–4 month old Wistar male rats fed on a standard cubed diet (M.R.C. diet 41). Rats were killed by cervical dislocation and livers perfused with ice cold 0·15 M sodium chloride and 0·25 M sucrose. Liver homogenates (20 per cent v/v) were then prepared in 0·25 M sucrose and fractions isolated by differential centrifugation. Mitochondria were sedimented at 10,000 *g* for 10 minutes and the pellets washed once by resuspension in one volume of 0·25 M sucrose. Following re-sedimentation (10,000 *g*, 15 minutes), these fractions were finally suspended in one volume of 0·25 M sucrose. Microsome fractions were isolated from mitochondrial supernatants by centrifugation at 105,000 *g* for 60 minutes. The supernatant (cell sap) fraction was removed and the microsome pellet re-suspended in 0·25 M sucrose. The cell sap and microsome fractions were then re-centrifuged (105,000 *g*, 60 minutes) and the microsome pellet finally re-suspended in 0·25 M sucrose (1 volume).

For assay, 200 mg. wet weight of liver or fractions from an equivalent weight of tissue were incubated aerobically in a medium containing 15 μmole glucose-6-phosphate (sodium salt); 0·3 μmole NADP (sodium salt), 300 μmoles nicotinamide, 150 μmoles potassium chloride and 150 μmoles sodium phosphate buffer (pH 7·4) in a total volume of 4·9 ml. Aminostilbene compounds (50 μg.) were added in ethanol (0·1 ml.) and incubated in air at 37° C. for 2 hours with constant gentle agitation. Metabolism was stopped by immersing flasks in boiling water for 5 minutes and metabolites were extracted into ether : ethanol (3 : 1 v/v mixture.

*Detection and estimation of aminostilbene metabolites*

Extracts containing aminostilbene metabolites were examined utilizing the following procedures.

*Paper chromatography* on Whatman No. 1 paper by the ascending technique using *tert*-butanol : formic acid : water (70 : 15 : 15 by volume) as developing solvent.

*Paper electrophoresis* on Whatman No. 1 paper using 30 per cent acetic acid as electrolyte. Electrophoresis was carried out for 12–18 hours with a potential gradient of 20 v/cm. (Current 3 ma./10 cm. wide-paper strip).

*Thin layer chromatography.*—Thin layer chromatography was carried out on 20 cm. × 20 cm. or 20 cm. × 10 cm. glass plates coated with a layer 0·2 mm. thick of silica gel G (Merck). Two developing systems were generally used; benzene : ethanol (9 : 1 v/v) and benzene : acetone (3 : 1 v/v) and these allowed separation of all the reference compounds (Table I).

TABLE I.—*Chromatography of 4-Aminostilbene Compounds*

| Compound | Paper chromatography Whatman No. 1. Developing solvent-butanol: formic acid: water (70 : 15 : 15) by volume Rf | Thin layer silica gel chromatography. Developing solvent: Benzene : ethanol (9 : 1 *v/v*) Rf | Benzene : acetone (3 : 1 *v/v*) Rf |
|---|---|---|---|
| 4-acetamidostilbene (AAS) | 0·86 | 0·21 | 0·48 |
| *N*-hydroxy-AAS | 0·83 | 0·20 | 0·30 |
| 4′-hydroxy-AAS | 0·78 | 0·08 | 0·28 |
| 3-hydroxy-AAS | — | 0·26 | 0·57 |
| 4-aminostilbene (AS) | 0·88–0·90 | 0·70 | 0·76 |
| 4′-hydroxy-AS | 0·75–0·79 | 0·19 | 0·49 |

Metabolites were visualized by direct observation of their blue fluorescence under ultraviolet light or by spraying with one of the following reagents:

(*a*) *p*-dimethylaminobenzaldehyde (1 %) in N HCl (Cramer, Miller and Miller, 1960).

(*b*) Diazotization with nitrous acid followed by coupling with $\beta$ naphthol (5 %) in 2N NaOH.

(*c*) Ferric chloride (0·3 %)—Potassium ferricyanide (0·3 %) (Smith, 1960).

*Quantitative estimation of N-hydroxy-AAS*

Determination of the concentration of *N*-hydroxy-AAS was carried out utilizing the colorimetric procedure of Sawicki, Stanley, Hauser, Elbert and Noe (1961) for detecting aromatic amines and imino heteroaromatic compounds.

Zones from paper chromatograms containing *N*-hydroxy-AAS were eluted with hot acetone and the extracts evaporated to dryness. The residues were redissolved in methanol (1 ml.) and aqueous 0·2 per cent 3-methyl-2-benzothiazolone hydrazone (1 ml.) BDH and 1·3 per cent ferric chloride (2 ml.) added. After 15 minutes, distilled water (2 ml.) was added and the mixture extracted with chloroform (4 ml.). The chloroform layer was then removed and its extinction measured at 675 m$\mu$ in a Unicam S.P. 600 spectrophotometer.

*Silicic acid chromatography of urinary metabolites*

Partition chromatography of urinary metabolites on silicic acid (Mallinckrodt, 100 mesh) was carried out essentially as described by Weisburger, Weisburger, Morris and Sober (1956). Samples were applied to the columns (2 × 38 cm.) in *tert*-butanol and elution carried out with the organic phase from a mixture of cyclohexane : *tert*-butanol : glacial acetic acid : water (16 : 4 : 2 : 1; by vol.). The eluate was collected in 5 ml. samples and examined for aminostilbene compounds by determination of the extinction at 320 m$\mu$ in a S.P. 600 spectrophotometer.

### RESULTS

*Urinary metabolism*

Paper chromatography of ether : ethanol (1 : 1) extracts of enzymatically hydrolysed urine from rats fed 4-acetamidostilbene (AAS) allowed detection, by comparison with reference compounds, of 4′-hydroxy-AAS (Rf 0·78) and 4-aminostilbene (Rf 0·88–0·90). Both metabolites exhibited blue fluorescence under ultraviolet light whilst the zone corresponding to 4′-hydroxy-AAS reacted with the ferric chloride–ferricyanide reagent to give a deep blue colour. Furthermore, the zone identified as 4-aminostilbene, like the authentic compound, reacted with nitrous acid and alkaline $\beta$-naphthol to produce a red dye.

Following extraction of the ether–ethanol extracts with dilute alkali, paper chromatography of the acidic metabolites enabled a third metabolite to be detected with an Rf (0·83) identical to that of authentic *N*-hydroxy-4-acetamidostilbene (*N*-hydroxy AAS). This zone and the authentic *N*-hydroxy-AAS reacted slowly with acidic $\beta$-dimethylaminobenzaldehyde and with the ferric chloride–potassium ferricyanide reagent. The metabolite also migrated at the same rate as the authentic *N*-hydroxy AAS (migration distance 11 cm.) following paper electrophoresis in 30 per cent acetic acid for 12 hours (potential gradient 20 v/cm.) and was well separated from the 4′-hydroxy-AAS derivative (migration distance 9·5 cm.).

Further characterization of the hydroxylated metabolites was obtained following silicic acid column chromatography. Bulk urine samples (1 L.) obtained from a group of 40 rats fed continuously on the low protein diet containing AAS were treated with $\beta$-glucuronidase and Taka diastase and metabolites extracted into ether : ethanol (3 : 1 v/v). The hydroxylated metabolites isolated by extraction with 0·5 N NaOH were then fractionated by partition chromatography on a silicic acid column using cyclohexane : *tert*-butanol : acetic acid : water (16 : 4 : 2 : 1 by volume) as eluting solvent (Weisburger, Weisburger, Morris and Sober, 1956). Two main acidic components were separated (Fig. 1) and the first fraction (A) eluted under conditions almost identical to those necessary for the separation of *N*-hydroxy-AAS. The second fraction (B) was less homogeneous, but comparison with the elution profiles of known compounds indicated the presence of 4′-hydroxy-AAS.

The identity of these two metabolites as *N*-hydroxy-AAS and 4′-hydroxy-AAS was further demonstrated by comparison of their paper chromatographic properties with the authentic compounds. Furthermore the ultraviolet absorption spectra of these metabolites (A and B) in the eluting solvent (cyclohexane : *tert*-butanol : acetic acid : water, 10 : 4 : 2 : 1) were identical with those of *N*-hydroxy-AAS ($\lambda$max 280 m$\mu$) and 4′-hydroxy-AAS ($\lambda$max 302 m$\mu$) respectively.

Quantitative studies indicated that initially, *N*-hydroxy-AAS was excreted in urine at a level of 9 $\mu$g./rat/24 hours (approximately 2 per cent of the administered dose). This is somewhat higher than the level (0·38–0·47 per cent) reported by Andersen, Enomoto, Miller and Miller (1964) following intraperitoneal administration of AAS. The level of *N*-hydroxy-AAS excreted decreased to approximately 4 $\mu$g./rat/24 hours after 4 weeks of AAS feeding, but then increased again so that after 12 weeks, the level was approximately equivalent to that initially observed.

In similar studies, it was demonstrated that *N*-hydroxy-AAS and 4′-hydroxy-AAS were excreted in urine following oral administration of 4-dimethylaminostilbene. With this compound, the level of *N*-hydroxy-AAS excreted was initially low (1·5 $\mu$g./rat/24 hours) ; (0·3 per cent of dose) but increased with continuous feeding to a level of 5 $\mu$g./rat/24 hours ; 1 per cent of dose after 12 weeks.

FIG. 1.—Partition chromatography of urinary metabolites of AAS on silicic acid.
Fraction A—Pool of fractions from tubes 7–20
Fraction B—Pool of fractions from tubes 30–45.
———————— Urinary metabolites.
– – – – – – – – – – – Reference compounds.

### *Classification of urinary metabolites*

Metabolites excreted in urine from rats fed AAS were fractionated into free compounds and sulphuric acid and glucosiduronic acid conjugates by alumina chromatography of ether : ethanol (3 : 1 v/v) extracts of urine using the procedure of Weisburger, Grantham, Morris and Weisburger (1961). The conjugates with sulphuric acid and glucosiduronic acid were then hydrolysed with Taka diastase and $\beta$-glucuronidase respectively and the liberated metabolites characterized by paper and thin layer chromatography. These studies showed that most of the *N*-hydroxy-AAS was present as a conjugate of glucosiduronic acid, whilst a small amount was excreted as a sulphuric acid ester (Table II). The other major metabolite detected, 4′-hydroxy-AAS, was present in the free form as well as conjugated with glucosiduronic acid and sulphuric acid. In addition, a small amount of 4-aminostilbene was detected as its glucosiduronic acid conjugate.

### *Metabolism of N-hydroxy-AAS*

Following oral administration of *N*-hydroxy-AAS to rats in the low protein diet, only a small amount of unchanged compound was excreted in urine (10 μg./24 hours ; approx. 2 per cent of administered dose) and this was present mainly as a glucosiduronic acid conjugate (Table II). The only other major metabolite detected was 4′-hydroxy-AAS and this was present both in the free form and conjugated to glucosiduronic acid. Additionally, a small amount of 4-amino-stilbene was detected as its glucosiduronic acid conjugate (Table II).

TABLE II.—*Urinary Metabolites of 4-Acetamidostilbene (AAS) and N-Hydroxy-AAS Following Oral Administration to Rats*

| Compound administered | Metabolites detected as:— Free compounds | Conjugates of Glucosiduronic acid | Conjugates of Sulphuric acid |
|---|---|---|---|
| AAS | 4′-hydroxy-AAS | 4′-hydroxy-AAS<br>N-hydroxy-AAS<br>4-aminostilbene (trace) | 4′-hydroxy-AAS<br>N-hydroxy-AAS |
| N-hydroxy-AAS | 4′-hydroxy-AAS<br>N-hydroxy-AAS | N-hydroxy-AAS<br>4′-hydroxy-AAS<br>4-aminostilbene (trace) | |

### *Metabolism studies* in vitro

Following incubation of AAS *in vitro* with rat liver homogenate in a system which permitted hydroxylation, in addition to unchanged compound, two blue fluorescent metabolites were detected by paper chromatography of ether : ethanol (3 : 1 v/v) extracts. These were identified by their staining reactions and by reference to known compounds as *N*-hydroxy-AAS (Rf 0·83) and 4′-hydroxy-AAS (Rf 0·78). The identities of these metabolites were further confirmed by comparison of their electrophoretic mobilities with those of the authentic compounds. Additionally, they were also characterized by thin layer chromatography (Table I). This technique also enabled the detection of two other metabolites which were identified by comparison of their Rfs and staining reactions with known compounds as 4-aminostilbene and 4′-hydroxy-4-aminostilbene (Table III).

When *N*-hydroxy-AAS was incubated *in vitro* with rat liver homogenate, the only metabolite detected was 4′-hydroxy-AAS together with unchanged compound (Table III).

TABLE III.—*Metabolism of 4-Aminostilbene Compounds by Rat Liver Homogenate**

| Substrate | Metabolites identified |
|---|---|
| 4-acetamidostilbene (AAS) | N-hydroxy-AAS<br>4′-hydroxy-AAS<br>4′-aminostilbene<br>4′-hydroxy-4-aminostilbene |
| N-hydroxy-AAS | 4′-hydroxy-AAS |

* Reaction mixtures and incubation conditions are described in the Methods section. The metabolites were identified on paper or thin layer chromatograms by comparison of their Rfs and colour reactions.

### *Intracellular localization of N-hydroxylating enzymes*

Comparison of the metabolic activity of subcellular fractions of rat liver demonstrated that the *N*-hydroxylating enzymes were located mainly in the microsome fraction, although co-factors in the cell sap were required (Table IV). Additionally, a small amount of *N*-hydroxy-AAS was detectable when AAS was incubated with the combined mitochondria-cell sap fractions.

TABLE IV.—*Metabolism of 4-acetamidostilbene (AAS) by Sub-cellular Fractions of Rat Liver*

| Cell fraction* | Metabolites detected |
| --- | --- |
| Microsomes . . . | None |
| Cell sap . . . | None |
| Microsomes + cell sap . | *N*-hydroxy-AAS<br>4′-hydroxy-AAS |
| Mitochondria . . | None |
| Mitochondria + cell sap. | *N*-hydroxy-AAS |

* Each cell fraction (equivalent to 200 mg. wet weight of liver) was suspended in the standard incubation system described in the Methods.

### DISCUSSION

The present studies indicate that an *N*-hydroxylated metabolite is formed following oral administration of 4-acetamidostilbene (AAS) in a low protein diet to adult Wistar rats, and excreted in urine as conjugates of glucosiduronic acid and sulphuric acid. This metabolite was also demonstrated in the urine of rats treated similarly with 4-dimethylaminostilbene although the levels in urine were lower than those detected in AAS-treated rats, presumably because of the additional requirement of *N*-demethylation of this compound. Andersen, Enomoto, Miller and Miller (1964) have also reported that AAS is metabolized to *N*-hydroxy-AAS following intraperitoneal administration to weanling rats. Furthermore, they were able to isolate sufficient of this metabolite from rats which received intraperitoneal injections of the less toxic 4-aminostilbene to permit its unequivocal identification by elementary analysis and by comparison of its physical properties with those of the authentic compound.

The other major metabolite detected in urine following oral administration of AAS or the *N*-hydroxy derivative was 4′-hydroxy-AAS, which was present in the free form and also as conjugates with glucosiduronic acid and sulphuric acid. Additionally, a small amount of 4-aminostilbene was detected as its glucosiduronic acid conjugate. These findings differ from those of Andersen, Enomoto, Miller and Miller (1964) where the major metabolite detected in urine following intraperitoneal injection of AAS or *N*-hydroxy-AAS was the *o*-hydroxy derivative, 3-hydroxy-AAS, although two unknown metabolites were detected one of which was identified tentatively as 4′-hydroxy-AAS. There were, however, a number of variations between the experimental systems used in the present studies and those of Andersen, Enomoto, Miller and Miller (1964), including differences between the strains of rats, the route of administration of compounds and probably most significantly, the nutritional status of the animals. Hence Weisburger, Grantham and Weisburger (1964) have recently reported differences in the metabolism of *N*-hydroxy-2-acetamidofluorene (*N*-hydroxy-AAF) in male and female rats, particularly with regard to the formation of ring hydroxylated derivatives. It has also been reported (Margreth, Lotlikar, Miller and Miller, 1964) that the levels of the urinary metabolites of AAF excreted by rats depended upon the dietary protein content. Thus the levels of one metabolite, 7-hydroxy-AAF, were 15 per cent and 24 per cent of the administered dose in rats maintained on diets containing casein at 50 per cent and 18 per cent respectively.

AAS was also converted to the *N*-hydroxy derivative by rat liver homogenate in a system which supports hydroxylation and the enzymes involved were shown to be contained in the microsome fraction. The other metabolites formed following incubation of AAS with rat liver homogenate were 4′-hydroxy-AAS, 4′-hydroxy-4-aminostilbene and 4-aminostilbene. The only metabolite detected following incubation of *N*-hydroxy-AAS in the rat liver homogenate system was 4′-hydroxy-AAS.

These findings are in agreement with recent studies demonstrating *N*-hydroxylation of carcinogenic aromatic amines by rat and rabbit liver homogenates or suitably fortified microsome fractions. Thus Irving (1962, 1964) and Booth and Boyland (1964) have detected *N*-hydroxy-AAF following incubation of AAF with rabbit liver microsomes in the presence of $NADPH_2$ and oxygen. Booth and Boyland (1964) also showed that 4-acetamidobiphenyl and *N*-acetylbenzidine were converted to the *N*-hydroxy metabolites by rabbit liver microsomes, whilst further metabolism of 4-acetamidobiphenyl resulted in hydroxylation at both the 4′- and 3- positions. The 4′-hydroxy metabolites were considered to arise by direct hydroxylation of 4-acetamidobiphenyl or the deacetylated derivative whereas *o*-hydroxylation was thought to arise from further metabolism of the *N*-hydroxylated metabolites.

Whilst there are some differences between the present studies and those of Andersen, Enomoto, Miller and Miller (1964) regarding the nature of the ring hydroxylated metabolites of AAS, it is doubtful whether these derivatives are directly involved in the carcinogenic process. Hence it has been demonstrated (Baldwin, Smith and Surtees, 1963*a*) that 4′-hydroxy-AAS is not carcinogenic when administered orally to rats under conditions whereby AAS and the *N*-hydroxy derivative are highly carcinogenic (Baldwin, Smith and Surtees, 1963*b*). Similarly, in studies still incomplete, Andersen, Enomoto, Miller and Miller (1964) have reported the inactivity of 3-hydroxy-AAS following subcutaneous injection into rats. These findings, together with the observation of high carcinogenic activity in *N*-hydroxy-AAS (Baldwin, Smith and Surtees, 1963*b* ; Andersen, Enomoto, Miller and Miller, 1964) suggest that *N*-hydroxylation is a critical metabolic change, necessary for carcinogenicity in the aminostilbenes, whereas ring hydroxylation represents a detoxication change.

Although evidence is accumulating that *N*-hydroxylation is involved in aromatic amine carcinogenesis, little is yet known about the nature of the cellular interactions with *N*-hydroxy metabolites. That arylhydroxylamines are highly reactive substances has been demonstrated by Boyland, Manson and Nery (1962), and Weisburger, Grantham and Weisburger (1964) have reported *in vivo* binding of *N*-hydroxy-AAF to tissue protein. Furthermore, the possibility of direct interactions with nucleic acids may be relevant since the mutagenic action of hydroxylamine has been ascribed to its interaction with cytosine bases in DNA (Freese, Bautz-Freese and Bautz, 1961) and Weisburger, Grantham and Weisburger (1964) have reported that the highly reactive deacetylated compound, *N*-hydroxy-2-aminofluorene combines with nucleic acid as well as protein.

Arylhydroxylamines have also been shown to chelate with metal ions (Miller, Enomoto and Miller, 1962 ; Weisburger, Grantham and Weisburger, 1963). Hence the possibility needs consideration of *in vivo* interactions of *N*-hydroxy-AAS with metal ions. Whilst the significance of such interactions remains to be elucidated, there is evidence that metal ions can influence carcinogenesis (Howell, 1958 Miller, Enomoto and Miller, 1962 ; Fare and Howell, 1964).

In the studies of Baldwin, Smith and Surtees (1963*b*) all the tumours induced following oral administration of AAS or the *N*-hydroxy-AAS arose in glands associated with the external auditory meatus and no tumours developed in liver. Investigations are now in progress to ascertain why the ear duct gland is sensitive and the liver refractory to aminostilbene carcinogenesis, particularly since it has been demonstrated that the liver is involved in *N*-hydroxylation of the carcinogen. Body distribution studies have demonstrated that 24 hours following a single intraperitoneal injection of $^{14}$C-labelled-AAS to rats, the level of radioactivity/g. wet weight of tissue in the ear duct gland ($5{\cdot}4 \times 10^4$ counts/minute) was comparable to that in liver ($4{\cdot}7 \times 10^4$ counts/minute). Moreover the radioactive material localized in the ear duct gland was found to persist (Baldwin and Romeril, unpublished observations). Clearly, these observations may be highly relevant in explaining tissue sensitivity to the carcinogenic action of aminostilbene compounds. The nature of the radioactive metabolites and the processes involved in their tissue distribution are now under investigation.

SUMMARY

1. *N*-hydroxy-4-acetamidostilbene (*N*-hydroxy-AAS) was identified as a urinary metabolite following oral administration of 4-acetamidostilbene (AAS) or 4-dimethylaminostilbene to rats. This metabolite was excreted mainly as a conjugate with glucosiduronic acid together with a small amount as a sulphuric acid ester.

2. The formation of *N*-hydroxy-AAS was also demonstrated following incubation of AAS with rat liver homogenate in a system which supported hydroxylation. The enzymes involved in *N*-hydroxylation were contained in the microsomes although co-factors in the cell sap fraction were necessary.

3. The other major urinary metabolite detected following oral administration of AAS was 4′-hydroxy-4-acetamidostilbene (4′-hydroxy-AAS) and this was excreted in the free form as well as conjugated with glucosiduronic acid and sulphuric acids. 4′-hydroxy-AAS was also the major urinary metabolite identified following oral administration of *N*-hydroxy-AAS to rats.

4. 4′-Hydroxy-AAS was also formed following incubation of AAS or *N*-hydroxy-AAS with the rat liver homogenate system. With AAS, the deacetylated metabolites 4-aminostilbene and 4′-hydroxy-4-aminostilbene were also identified.

5. The results, together with the reported lack of carcinogenic activity of ring hydroxylated aminostilbene compounds, supports the concept that *N*-hydroxylation is a necessary stage for carcinogenic action.

We wish to thank Professor M. W. Partridge, Department of Pharmaceutical Chemistry, University of Nottingham, for advice and assistance in the synthesis of aminostilbene compounds. During the course of these investigations we learned that similar studies were being carried out by Professor E. C. Miller and associates, University of Wisconsin, U.S.A. We are grateful to Professor Miller for the opportunity to exchange information regarding these studies. This work was supported by a block grant from the British Empire Cancer Campaign for Research.

### REFERENCES

ANDERSEN, R. A., ENOMOTO, M., MILLER, E. C. AND MILLER, J. A.—(1964) *Cancer Res.*, **24**, 128.

BALDWIN, R. W., SMITH, W. R. D., AND SURTEES, S. J.—(1963*a*) *Rep. Brit. Emp. Cancer Campgn*, **41**, 428.—(1963*b*) *Nature, Lond.*, **199**, 613.
BELL, F., KENYON, J. AND ROBINSON, P. H.—(1926) *J. chem. Soc.*, 1239.
BOOTH, J. AND BOYLAND, E.—(1964) *Biochem. J.*, **91**, 362.
BOYLAND, E., DUKES, C. E. AND GROVER, P. L.—(1963) *Brit. J. Cancer*, **17**, 79.
*Idem*, MANSON, D. AND NERY, R.—(1960) *Rep. Brit. Emp. Cancer Campgn*, **38**, 52—(1962) *J. chem. Soc.*, 606.
CRAMER, J. W., MILLER, J. A. AND MILLER, E. C.—(1960) *J. biol. Chem.*, **235**, 885.
ELSON, L. A.—(1952) *Brit. J. Cancer*, **6**, 392.
FARE, G. AND HOWELL, J. S.—(1964) *Cancer Res.*, **24**, 1279.
FREESE, E., BAUTZ-FREESE, E. AND BAUTZ, E.—(1961) *J. molec. Biol.*, **3**, 133.
HADDOW, A., HARRIS, R. J. C., KON, G. A. R. AND ROE, E.—(1948) *Phil. Trans.*, **241**, 147.
HOWELL, J. S.—(1958) *Brit. J. Cancer*, **12**, 594.
IRVING, C. C.—(1962) *Biochim biophys. Acta.*, **65**, 564.—(1964) *J. biol. Chem.*, **239**, 1589.
MARGRETH, A., LOTLIKAR, P. D., MILLER, E. C. AND MILLER, J. A.—(1964) *Cancer Res.*, **24**, 920.
MASSERANI, A.—(1957) *Farmaco*, **12**, 380.
MILLER, E. C., MILLER, J. A. AND HARTMANN, H. A.—(1961) *Cancer Res.*, **21**, 815.
MILLER, J. A., ENOMOTO, M. AND MILLER, E. C.—(1962) *Ibid.*, **22**, 1381.
*Idem*, WYATT, C. S., MILLER, E. C. AND HARTMANN, H. A.—(1961) *Ibid.*, **21**, 1465.
SACHS, F. AND SACHS, L.—(1905) *Ber. dtsch. chem. Ges.*, **38**, 511.
SAWICKI, E., STANLEY, T. W., HAUSER, T. R., ELBERT, W. AND NOE, J. L.—(1961) *Analyt. Chem.*, **33**, 722.
SMITH, I.—(1960) 'Chromatographic and Electrophoretic Techniques', London (Pitman), 1, 324.
TROLL, W. AND NELSON, N.—(1961) *Fed. Proc.*, **20**, 41.
UEHLEKE, H.—(1963) *Biochem. Pharmacol.*, **12**, 219.
WEISBURGER, E. K., GRANTHAM, P. H. AND WEISBURGER, J. H.—(1964) *Biochemistry*, **3**, 808.
WEISBURGER, J. H., GRANTHAM, P. H., MORRIS, H. P. AND WEISBURGER, E. K.—(1961) *Cancer Res.*, **21**, 949.
*Idem*, GRANTHAM, P. H. AND WEISBURGER, E. K.—(1963) *Biochem. Pharmacol.*, **12**, 179.
*Idem*, WEISBURGER, E. K., MORRIS, H. P. AND SOBER, H. A.—(1956) *J. nat. Cancer Inst.*, **17**, 363.